# Psychiatrists’ practices on monitoring cardio-metabolic adverse effects: a narrative review

**DOI:** 10.1192/j.eurpsy.2025.1196

**Published:** 2025-08-26

**Authors:** D. Dos Santos, A. C. Silva, F. Miranda, M. Araújo

**Affiliations:** 1 Department of Psychiatry and Mental Health, Hospital Garcia de Orta, Almada, Portugal

## Abstract

**Introduction:**

Several medications in use in the psychiatry field, particularly in severe mental illness (SMI), are associated with weight gain and other cardio-metabolic side effects and are usually prescribed long-term. Extensive research has been conducted on the contribution of these effects to poorer physical health and a 10- to 20-year mortality gap in patients with SMI. Many factors have been reported, such as patients’ lifestyle behaviours, lower search for medical care, delayed diagnosis and treatment, diagnostic overshadowing by mental illness, and stigma towards this population. In this context, psychiatrists may have a role in monitoring medication’s side effects their role is still to be defined.

**Objectives:**

To access usual practices and barriers on cardio-metabolic monitoring of patients by psychiatrists.

**Methods:**

We conducted a narrative review on general psychiatrists’ practices of cardio-metabolic monitoring. A search was conducted in Pubmed and PsycNet using the keywords “practice”, “assessment”, “weight gain”, “metabolic”, and “psychiatrist”. Further papers were included after references and citations review of relevant articles. Papers focused exclusively on guidelines or on border programs (such as first psychotic episode protocols) were excluded.

**Results:**

Most of the research dedicated to reporting psychiatrists’ practices regarding monitoring of cardio-metabolic side effects focused on patients treated with long-term antipsychotics. Despite knowledge and concern about these side effects being transversally reported, it doesn’t always translate into clinical practice, and practices are widely diverse. Many factors were appointed as explanations; the most commonly reported were training limitations, poorer physical examination skills, underestimation of side effects, overshadowing of co-occurring physical disease by mental symptoms, lack of scientific consensus amongst different specialties resulting in contradictory guidelines, fragmentation of care across different healthcare providers and specialties, and the still undetermined role of psychiatrists in diagnosing and treating cardio-metabolic complications.

**Conclusions:**

Despite knowledge and awareness of cardio-metabolic side effects are almost transversal in psychiatrists’ practice and translated into the selection of treatments, management of these complications still needs to be put into action. Some strategies may be introduced, such as improving medical training and including this training in residency programs, developing consensual and multidisciplinary guidelines for the assessment and treatment of these side effects, defining the responsibility and improving the coordination of different health care providers in a community setting.

**Disclosure of Interest:**

None Declared

